# Application and progress of psychological sand table play therapy in children's rehabilitation

**DOI:** 10.3389/fped.2026.1790000

**Published:** 2026-06-16

**Authors:** Xuemei He, Chonyang Din, Xueyan He, Yun Liu

**Affiliations:** 1 Department of Rehabilitation, Kunming Children’s Hospital, Kunming Medical University, Kunming,Yunnan, China; 2 Women’s Psychiatric Department, Second People’s Hospital of Pu ‘er, Pu 'er, Yunnan, China

**Keywords:** children's rehabilitation, clinical efficacy, digital development, non-verbal intervention, sand table play therapy

## Abstract

This article provides a comprehensive review of the application and recent advances of sandplay therapy in pediatric rehabilitation. It critically examines empirical evidence supporting its clinical efficacy in alleviating psychological trauma, ameliorating emotional and behavioral dysregulation, fostering social competence, and facilitating cognitive development. Furthermore, the review evaluates current implementation patterns, identifies key methodological and practical limitations, and outlines emerging directions for research and clinical integration—thereby offering a robust theoretical and evidence-informed foundation for refining and scaling sandplay interventions in child rehabilitation settings.

## 1 Introduction

Sandplay therapy, also known as sandbox therapy, is a form of therapy where, under the guidance of a therapist, the client freely selects miniature figures from a shelf and arranges them in a specially designed container filled with fine sand to create scenes. The therapist then uses Jung's theory of “archetypes” to analyze the client's work (1). As a non-verbal form of psychotherapy, although it riginated from the theories of the psychoanalytic school, it is capable of integrating and applying the three major psychotherapy schools of psychoanalysis, behaviorism, and humanism (2). Traditional language-oriented therapy has limited effects on young children or those with language limitations. Psychological sandplay therapy, through symbolic expression, provides children with a form of “subconscious language”, which is more suitable for the characteristics of children's mental development (3).

During the growth process, children may encounter various situations that hinder their physical and mental development, such as neurodevelopmental disorders like autism spectrum disorder and attention deficit hyperactivity disorder (ADHD), emotional disorders like anxiety and depression, behavioral problems like aggression and withdrawal, psychological trauma caused by family changes or school bullying, and physical diseases like cerebral palsy that lead to delayed physical and mental function development. These situations not only interfere with children's cognitive development, emotional regulation and social interaction skills, but also may have adverse effects on their personality formation and long-term quality of life. Psychological sandplay therapy, as a psychological treatment technique, provides children with a non-verbal and symbolic expression space, which is conducive to exploring the psychological content in their subconscious and touching the pre-verbal level deep in their hearts (4).

In recent years, sandplay therapy has been increasingly applied in clinical psychological assessment and treatment. Richards stated that school children can externalize their trauma due to the safe environment created in sandplay therapy, effectively reducing their social withdrawal level. Sandplay therapy requires little language skill and allows children to express themselves or respond nonverbally through sandplay creations (5). Judan Tan conducted a randomized controlled trial on 60 children with chronic diseases. The results showed that sandplay therapy could alleviate the anxiety, withdrawal and social behavior problems of school-aged children with chronic diseases, and also relieve the anxiety and depression symptoms of their caregivers (6). Lu, L applied sandplay to school children with autism spectrum disorder. The results suggested that sandplay successfully increased the children's language expression levels, their participation in social activities and their symbolic, voluntary and creative play performance (7).

However, the introduction of sandplay therapy to China is relatively recent, and it is still in its early development stage. Currently, most of the research on children's sandplay in China is case studies, and some experiments have the problem of insufficient sample size, which cannot effectively prove the effectiveness of sandplay therapy. Moreover, most of the interventions for children focus on psychological and behavioral problems in normal children; there is still a lack of analysis and intervention for the psychology of special children, such as those with chronic diseases or disabilities (4).This article summarizes the application and progress of psychological sandplay therapy in children's rehabilitation, aiming to systematically review the current application status and research progress of psychological sandplay therapy in the field of children's rehabilitation, clarify its effects, advantages, and existing problems in different types of children's rehabilitation, and provide theoretical references and practical guidance for subsequent clinical practice. At the same time, it is hoped that this can draw more attention from the academic community to the psychological intervention of special children, promote the development of related research towards a larger scale and standardization, further expand the application boundaries of psychological sandplay therapy, improve the quality of service and social value of psychological sandplay therapy in the field of children's rehabilitation, and contribute to the mental health and all-round development of children.

## 2 Overview of psychological sand table game therapy

### 2.1 Theoretical basis

Sand table therapy originates from Jung's analytical psychology and integrates the concept of “unity of body and mind” in Eastern philosophy, emphasizing the awakening of individual self-healing power through symbolic games (8). Kalff proposed the theory of “freedom and protected space”, believing that when children reconstruct their inner world in the sand table, the therapist's non-judgmental companionship can enhance their sense of security and thus promote self-healing (9). Its development history can be traced back to the “floor game” in the early 20th century. Later, it was integrated with Lowenfeld's “world technology” and Kalff and finally formed a treatment system with self-development as the core (10, 11).

### 2.2 Treatment process

In a standard psychological sand tray therapy room, there is a sand tray filled with fine sand and a large number of miniature models such as characters, animals, buildings, transportation, etc. The therapist guides children to select models and create scenes in the sand table freely. Children can arrange and adjust the sand table according to their wishes. Throughout the process, the therapist carefully observes and records the details of the child's behavior, the model of choice, the position and order of placement, and uses this information to interpret the child's psychological state.

The therapeutic process typically unfolds in three stages:

(a) Free creation: Children's independent selection of models to construct scenarios.
(b) Observation records: Therapists focus on model selection, spatial layout, and emotional responses.
(c) Guide and reflect: Help children connect sand table imagery and real experience through asking questions (12) Research shows that the initial sand table (first creation) can reflect children's adaptability to treatment, the relationship between consciousness and unconsciousness, and potential problem-solving paths (13).

## 3 Application of psychological sand table play therapy in children's rehabilitation

### 3.1 Emotional disturbance

Children with emotional disorders (such as anxiety, depression, post-traumatic stress disorder, etc.) often have limited language expression abilities or are emotionally repressed, making it difficult for them to directly express their inner feelings. The non-verbal nature of psychodrama therapy precisely meets this need.

Multiple clinical studies have verified the intervention effect of sandplay therapy on children with emotional disorders: For instance, a randomized controlled trial targeting children aged 7–14 with anxiety disorders showed that after 8 weeks of sandplay intervention, the total score of the Anxiety Disorders Screening Scale (SCARED) for the experimental group significantly decreased (17.30 ± 4.47 vs. 34.30 ± 4.47, *P* < 0.001), and the score of the Emotional Stability N Scale improved significantly (14). PingWang et al. [applied sandplay therapy to a deaf child (12 years old) with social anxiety disorder. This study adopted a reverse experimental design. The results showed that sandplay therapy could make up for the language expression deficiencies of the deaf child and effectively alleviate the anxiety condition (15).Canrui Chen took the sandplay therapy sandboxes created by 29 bereaved students who voluntarily participated in the activity at a relief center in the disaster-stricken area as the experimental group, and the sandboxes made by 16 ordinary students who voluntarily participated in the sandplay activity at a primary school in Guangzhou as the control group. He analyzed the characteristics of the sand drawings and sand tools used by the 29 students who suffered from bereavement due to the earthquake. The proportion of students in the bereaved group who expressed trauma themes in the sandplay game was higher than that in the control group, suggesting that sandplay therapy can reveal the psychological problems of the bereaved students after the earthquake (16).

Lee MB's research indicates that sandplay therapy can significantly reduce the depression scores of children who are victims of cyberbullying (a 39.7% decrease on the CDI scale) and greatly reduce suicidal ideation (a 77.8% decrease on the SIQ-JR scale) (17). Jianhua Ding conducted a randomized controlled trial (*n* = 75), and the results indicated that the treatment efficacy of the combined group of sandplay therapy and cognitive behavioral therapy was 92.5%, significantly higher than that of the sandplay therapy group alone (74.2%) (*P* < 0.05); after treatment, the scores of SAS and SDS in the combined group decreased by 34.6% and 30.9% respectively, and the treatment compliance (92.5% vs. 71.4%) and parental satisfaction (92.5% vs. 74.2%) were both significantly improved, suggesting that cognitive behavioral therapy, by correcting cognitive biases and combining non-verbal expressions of sandplay, can synergistically alleviate anxiety and enhance the sustainability of treatment (18).

The psychological sandplay therapy, with its non-verbal nature, provides a safe platform for emotional release and self-expression for children with language limitations or suppressed emotions who suffer from emotional disorders. From anxiety disorders, depression to post-traumatic stress disorder, from ordinary children to special groups (such as deaf children, children with central nervous system tumors, students who lost their parents in the earthquake, and children who suffered from cyberbullying), numerous clinical studies have confirmed its significant intervention effects. This indicates that psychological sandplay therapy has important clinical value in the field of children's emotional disorder rehabilitation. In the future, by integrating deeply with multidisciplinary therapies, it can better adapt to the rehabilitation needs of different children and promote its wide application in the practice of children's emotional rehabilitation.

### 3.2 Autistic disorder

The core characteristics of children with autism spectrum disorder include social communication difficulties, stereotyped behaviors and abnormal sensory perception. Their language and non-verbal communication abilities are all limited, and traditional talk therapy is difficult to implement.

Autistic children often exhibit a distinct “order preference” in sandplay games. For instance, they tend to arrange sand toys or objects in symmetrical patterns. This pattern directly reflects their inherent stereotypical thinking characteristics and may provide them with a controllable means of self-expression. The psychological sandplay therapy, with its non-verbal, symbolic and low social-pressure characteristics, provides autistic children with a possible channel for emotional expression and social interaction.

A number of studies have explored the intervention effect of sandplay therapy on children with autism: Liang Liang conducted a randomized controlled trial on 90 children with autism, and the results showed that the outcome of the treatment for autism spectrum disorders in the observation group was 44 (97.78) (with marked improvement in 36 cases and effective improvement in 8 cases), which was higher than that of the control group 31 (68.89) (with marked improvement in 19 cases and effective improvement in 12 cases), *P* < 0.05. This suggests that sandplay therapy for autism spectrum disorders can effectively improve the core symptoms of the children and shorten the treatment time (9).

Jieling Zeng used sandplay therapy to conduct psychological intervention on children with autism. The results showed that sandplay therapy could effectively alleviate the symptoms of autism (19). Enyao Li observed the effect of sandplay games combined with comprehensive intervention (such as sensory integration training, speech therapy, etc.) in treating children with autism. The results showed that the combined treatment could effectively alleviate the symptoms of autistic children, but it was unable to distinguish the independent contribution of sandplay games (20). Yanxia Wang randomly divided preschool children with autism spectrum disorder into cross-group sandplay group and individual sandplay group. After 12 weeks of intervention, the core symptoms of both groups of children improved, and the improvement in social interaction aspect of the group sandplay was better than that of the individual sandplay (*P* < 0.05) (21). Guiping Liu conducted sandplay therapy for autistic children and found that sandplay games were helpful in improving the communication ability and imagination of the children (22). Guo J and Li conducted a randomized controlled trial on 90 autistic children aged 4–6 years. Their research proposed the innovative Image-Sandplay Therapy, which combines visual imagery guidance with sandplay creation. This therapy not only significantly improved the life satisfaction of autistic children (with a 13.5% increase in SWLS scale score), but also significantly reduced the frequency of problem behaviors (with a 28.3% decrease in ABC scale score) (23).

In conclusion, psychodrama therapy provides a non-verbal expression channel for autistic children with limited language communication. Current studies suggest that it has certain effects on core symptoms and emotional behaviors. The group form may be superior to the individual form in some aspects. However, most studies have limitations such as small sample size, lack of randomization and active control groups, short intervention period, and missing follow-up. In the future, larger sample, multi-center, strictly randomized controlled trials should be conducted, and the synergistic effects of psychodrama therapy with behavioral intervention, family training, etc. should be explored to clarify its independent and incremental efficacy in the rehabilitation of autism.

### 3.3 Attention deficit hyperactivity disorder (ADHD)

ADHD is mainly characterized by inattention, hyperactivity-impulsivity, and difficulties in emotional regulation. These symptoms often lead to learning difficulties, strained peer relationships, and family conflicts. Although traditional drug treatment can alleviate the core symptoms, it has limited effect on improving emotional problems and behavioral management skills, and some children may experience drug intolerance. The psychological sandplay therapy, through structured tasks, time-limited creation and symbolic expression, may help train the attention and self-control abilities of children with ADHD.

Children with ADHD mainly exhibit symptoms such as inattention, hyperactivity-impulsivity, and difficulty in emotional regulation. These symptoms often lead to learning difficulties, strained peer relationships, and family conflicts. Although traditional drug treatment can alleviate the core symptoms, it has limited effects on emotional problems and behavioral management skills, and some children may have drug intolerance. Psychological sandplay therapy, through structured tasks, time-limited creation, and symbolic expression, may help train the attention and self-control abilities of children with ADHD.

A number of clinical studies have explored the intervention effect of sandplay therapy on children with ADHD: Jie Xu conducted a case study of a child with ADHD who received sandplay therapy (sandplay treatment), and found that after the intervention, the child's hyperactivity symptoms decreased, self-control ability improved, and interpersonal relationships and parent-child relationships were also enhanced (24). Wenyu Yu conducted sandplay psychological and behavioral intervention for children with ADHD. The results showed that after the intervention, the hyperactivity symptoms of the children significantly decreased and their self-control ability significantly improved (25).

Yanling Zhang conducted a randomized controlled trial on 120 school-age children with ADHD. The observation group (*n* = 60) received methylphenidate hydrochloride combined with sandplay therapy (once a week for 8 weeks), while the control group (*n* = 60) only received medication treatment. The results showed that the scores of oppositional behavior, hyperactivity-impulsivity, and inattention symptoms in the observation group were reduced by 23.4% to 31.7% compared to the control group (*P* < 0.05). The anxiety (SCARED) and depression (DSRSC) scores decreased by 39.1% and 44.6% respectively, and the attention quotient increased by 27.5% (*P* < 0.05) (26). Xueqin Li observed the impact of sandplay therapy combined with sensory integration treatment on the cognitive functions of children with ADHD. The results showed that the combined treatment was helpful in improving the cognitive functions of the children (27). Roesler C believes that sandplay therapy has certain therapeutic effects on mental disorders such as ADHD, and may be superior to traditional psychological treatment (8).

In conclusion, the combination of psychological sandplay therapy and medication treatment shows superior short-term effects in improving the core symptoms and negative emotions of children with ADHD compared to medication alone. It also helps to enhance attention and behavioral management abilities. However, the current studies still have deficiencies in blind implementation, sample size estimation, long-term follow-up, and the setting of active control. In the future, it is necessary to further explore the appropriate dosage, optimal intervention frequency, and mechanism of sandplay as an independent intervention or adjunctive treatment, and to verify its independent efficacy and long-term sustainability through multi-center, large-sample, and strictly randomized controlled trials.

### 3.4 Behavioral problems

Children with behavioral problems often exhibit aggressive behaviors, oppositional defiance, rebellious mentality, peer conflicts and conduct issues. These problems are often closely related to underlying emotional distress, family environment or traumatic experiences. Traditional verbal counseling is difficult to penetrate into the inner world of children, while sandplay therapy, through symbolic expression and projection mechanisms, may help children externalize conflicts and understand the emotional motivations behind their behaviors.

A number of clinical studies have explored the intervention effect of sandplay therapy on children with behavioral problems: Jiajun Tong applied short-term sandplay therapy to treat the behavioral problems of preschool children (aged 4–5 years), and the results showed that the progress made by the cases in the experimental group during the playroom sessions smoothly transferred to their daily lives (28). Wei Shi conducted sandplay therapy for neglected children. The results showed that the children's emotions improved, their self-confidence was restored, and their neglected social status was enhanced (29). Jing Chen used group sandplay therapy to intervene with 30 children (aged 6–13) who had behavioral problems. A pre-post control design was adopted. The results showed that group sandplay therapy could effectively promote the psychological growth of the problem children. However, this study did not set up a control group, and the efficacy assessment lacked objective behavioral indicators (30).

The Sandplay Therapy Research Group of the Juvenile Offender Correction Center in Shanghai (31) analyzed the characteristics of the sandplay works of 12 juvenile offenders and found that the themes of their sandplay were not rich, the content lacked vitality, and their personal roles were chaotic. This suggests that sandplay may be used for the assessment of behavioral problems. Kwak conducted a school sandplay group therapy for 284 ordinary primary and secondary school students (grades 4–6) over 10 sessions, each lasting 40 min. Using a pre-post control design, the results showed that the children's scores for oppositional and defiant behavior decreased from 46.54 to 44.77 (*P* = 0.045), and their conduct problems decreased from 48.59 to 45.63 (*P* = 0.007). This suggests that group sandplay therapy has a certain positive effect on behavioral problems (32).

In conclusion, psychodrama therapy provides a low-threat emotional expression space for children with behavioral problems, helping them to vent negative emotions and externalize internal conflicts. Existing studies suggest that it may have an improving effect on aggressive behavior, peer relationships, oppositional defiant behavior, and conduct problems. However, current research on sandplay intervention for children with behavioral problems mostly consists of small sample case studies or pre-post designs, lacking strict randomized controlled trials and long-term follow-up evidence. In the future, more rigorous controlled studies should be conducted to distinguish the differences in the responses of different types of behavioral problems (such as externalizing vs. internalizing) to sandplay therapy, and to explore the combined application effects of sandplay games with behavioral correction, parent training, etc.

### 3.5 Special children's intervention (language barriers, deaf children, intellectual disabilities, etc.)

Language barriers, deaf children, children with intellectual disabilities and other children with communication limitations often have their psychological needs and emotional problems overlooked or misjudged due to the narrowness of their expression channels. The non-verbal nature of psychological sandplay therapy makes it have unique application value among special children groups. It can make up for the deficiency of language communication through visual, tactile and symbolic expressions.

A number of studies have explored the applicability of sandplay therapy in special children: Wang Ping et al. conducted sandplay therapy on a 12-year-old deaf child with social anxiety disorder using the reverse experimental design (A-B-A-B), and the results showed that the score of the Social Anxiety Scale (LSAS) decreased by 52% after the intervention (15). Sakurai Yoko conducted a comparative study before and after sandplay therapy for all students in a school with severe language disorders in Australia, and found that the children's language communication ability and interpersonal interaction ability significantly improved after the intervention (56). Risheng Zhang established a sandplay therapy room at the deaf-mute school and conducted long-term psychological clinical practice there. He found that sandplay therapy was helpful in improving the emotional expression and peer interaction of deaf children (33). Jie Li conducted a sandplay game analysis study on the sexual behavior issues of one child with intellectual disability. The results showed that sandplay games were helpful in revealing the inner conflicts of children with intellectual disabilities and providing intervention entry points. However, this study was a case analysis and lacked quantitative evaluation and comparison (34). Additionally, Guoping Fan explored the application of sandplay games in kindergarten education practice, finding that this technique had a good transformation effect on some children with unhealthy psychological characteristics. However, the research subjects were not a specific group of special children, and the evaluation criteria were subjective (35).

In conclusion, the psychological sandplay therapy, with its non-verbal and multi-sensory interactive features, provides a feasible psychological intervention approach for special children such as those with language disorders, deaf children, intellectual disabilities, and selective mutism. The existing studies are mostly case reports or pre-post designs, with small sample sizes and lacking strict controls, resulting in low evidence quality. In the future, larger sample randomized controlled trials should be conducted, standardized sandplay intervention programs suitable for different types of disorders should be developed, and integrated models of sandplay games with language rehabilitation training and special education should be explored.

### 3.6 Child cerebral palsy

Cerebral palsy (abbreviated as CP) refers to the non-progressive brain injury that occurs before, during, or within one month after a child's birth, causing motor disorders and abnormal postures. It is often accompanied by intellectual, language impairments and behavioral abnormalities. The prevalence of cerebral palsy in China is approximately 2‰, with currently 4–5 million children affected and a disability rate of 42%–45%, imposing a heavy burden on families and society (36). At present, the rehabilitation of cerebral palsy mainly focuses on the recovery of physical functions, while the attention paid to psychological development is relatively weak. Children with cerebral palsy generally have psychological and behavioral problems such as anxiety, depression, and social withdrawal, and effective psychological intervention methods urgently need to be explored.

Tao Wang conducted a randomized controlled trial on 90 children with cerebral palsy aged 3–5 years. The experimental group received 10 sessions of group sandplay therapy in addition to the conventional rehabilitation treatment. The results indicated that the scores of social withdrawal, depression, aggression, and destructive behavior in the experimental group significantly decreased and were lower than those in the control group (*p* < .001) (37). And Tao Wang pointed out in the review that the non-verbal and symbolic nature of sandplay therapy may be suitable for the expression needs of children with cerebral palsy. The combined application of conventional physical rehabilitation therapy and sandplay therapy can improve the abnormal behaviors of children with cerebral palsy and awaken their self-healing ability. However, this review did not include specific original research data, and the related conclusions need to be verified (38).

In conclusion, the application of psychological sandplay therapy in the psychological rehabilitation of children with cerebral palsy is still in the stage of theoretical exploration. The existing evidence is merely descriptive summaries or citations of literature, lacking published randomized controlled trials, cohort studies, or systematic case reports. Given that children with cerebral palsy commonly have psychological problems and have limited channels for expression, sandplay therapy has potential application value. However, this assumption still needs to be verified through rigorous original research. In the future, feasibility studies and pre-experiments should be conducted to clarify the actual effects of sandplay therapy on the emotional and behavioral problems of children with cerebral palsy, as well as the appropriate intervention plans and safety.

## 4 Advantages and challenges of psychological sand table play therapy in children's Rehabilitation

### 4.1 Advantages

#### 4.1.1 Non-verbal

For children with limited linguistic expression skills or those unwilling to communicate verbally, sand table games provide a non-verbal way of expression. Through the children's play process and the images presented on the sand table, therapists can gain an in-depth understanding of the children's psychological characteristics and provide targeted guidance and help (39), which is more suitable for children with language delay.

#### 4.1.2 Hobby

The sand table game is enjoyable and highly appealing to children. Children are more likely to receive and participate in sand table play therapy more than traditional conversational therapy. They can relax their body and mind during the game, allowing them to show their inner world more naturally. Experiments have found that children's fear of medical staff is reduced after sand table playtreatment, enabling them to cooperate more effectively with medical staff for rehabilitation treatment.

#### 4.1.3 Creativity

Children can fully harness their creativity through the sand table game and build their world. Sand table games can help children express their true feelings freely, face internal conflicts bravely, and promote the development of self-integration. Experiments show that children initially pay more attention to their own issues at the beginning and tend to resist to contact with or being rejected by the outside world. After the therapist provides effective guidance to them, children's bad psychology is reduced (17).

### 4.2 Challenges

#### 4.2.1 The efficacy assessment relies on the subjective experience of the therapist

This may lead to inconsistent assessment standards, large fluctuations in results, a lack of repeatability and objective measurement indicators, thereby affecting the scientificity and reliability of treatment decisions.

#### 4.2.2 Limited effect on children with severe cognitive impairment

Due to the severe damage to these children's cognitive functions, traditional intervention methods often fail to effectively improve the core symptoms. Therefore, more targeted comprehensive therapies or auxiliary technologies need to be explored.

#### 4.2.3 The professional standards for therapists are very high

Psychoanalytic sandplay therapy requires therapists to have a solid foundation in child psychology and psychoanalytic theory, as well as extensive experience in interpreting sandplay works. They must be able to accurately capture the emotional expressions and psychological needs behind the logical choices and placements of children's sand toys. If therapists lack systematic professional training, they are prone to misinterpret the symbolic meanings of the sand toys or the behavioral motivations of children, thereby affecting the targeted and effective nature of the treatment plan.

#### 4.2.4 The treatment process is long and costly

Sandplay therapy usually requires multiple consecutive interventions to gradually improve a child's psychological state. The long-term treatment process not only increases the time and financial burden on the family, but also may lead to treatment interruptions due to the limited attention span of the child or the lack of patience of the parents, thereby reducing the rehabilitation effect.

#### 4.2.5 Lack of standardized operation guidelines

Currently, there are no unified industry standards for the operation procedures of psychological sandplay therapy, the configuration standards of sand materials, and the frequency of treatment. The practices of different institutions or therapists vary significantly, which hinders the stable replication of treatment effects and the promotion of the industry.

## 5 The development trend of psychological sandplay therapy in children's rehabilitation

### 5.1 Fusion with other treatment methods

In the future, psychological sand table play therapy will increasingly be combined with other treatment methods, such as cognitive behavioral therapy, family therapy, and so on. For example, when performing sand table play therapy for children, combined with some techniques from cognitive behavioral therapy, cognitive behavioral therapy is a comprehensive intervention model with strong interest and no somatotoxic side effects, which can effectively improve their social functional defects. In cases where family factors influence a child's psychology, family therapy can be incorporated into sand table play therapy, allowing family members to participate in sand table creation, and improve family relationships and communication models. Emerging approaches also explore combining sand tables with neurofeedback and biomarker monitoring, for instance adjusting the difficulty of sand table tasks through real-time EEG data to achieve personalized rehabilitation plans (40). Additionally, promoting the deep integration of sand tables with structured interventions such as CBT and mindfulness could establish aa comprehensive “cognition-behavior-emotion” treatment model (41).

### 5.2 Digital development

With the continuous advancement of technology, digital sand table games may gradually emerge. Through virtual reality (VR) or augmented reality (AR) technology, more diverse sandbox scenarios and models can be created. This digital sand table offers children a new experience, and facilitates therapists in collecting and analyzing data, including children's operating behavior and time spent on virtual sand table through software. When combined with VR development of dynamic sand tables, clinical trials in 2023 showed that VR sand tables can quantify children's attention distribution (the accuracy of eye tracking data reaches 89%) (42); based on neuroimaging technology (such as fMRI), it analyzes the reshaping effect of sand table therapy on the connection between the default mode network (DMN) and the executive control network (ECN) in children with ADHD (43).

### 5.3 Professional and standardized development

The application of psychological sand table play therapy in children's rehabilitation will become increasingly specialized and standardized. The relevant training system will be more complete, requiring therapists to undergo rigorous training and assessment to master solid theoretical knowledge and practical skills. At the same time, the treatment process and evaluation criteria will be further refined to ensure the quality and effectiveness of the treatment. The establishment of a “Consensus on Clinical Practice of Children's Sand Tail Treatment” and the standardization of operating procedures and efficacy scales (such as the SCTAS evaluation system) will provide a solid basis for quality assurance.

Moreover, empirical evidence supports the wider adoption of sand table interventions in broader contexts. For example, a large sample study of School Sand Table Group Therapy (SSGT) in Korea (*n* = 284) showed that it significantly improved the self-esteem of the general child (5.3% increase in the KCYP scale) and a reduction in depression symptoms (19.2%) (44). These results highlight its potential for integration into school-based mental health services, offering accessible, engaging, and effective support for children's psychological well-being.

### 5.4 The application prospects of psychological sandplay in children's psychological assessment

The non-verbal nature of the psychological sandplay precisely compensates for the limitations of children's insufficient language expression ability. Through dimensions such as sand toy selection preferences, sand painting spatial layout, and interactive patterns during the creative process, it can comprehensively capture the subconscious psychological state of children. In the future, with the penetration of digital technology, AI-driven sandplay assessment systems will be able to automatically analyze data such as sand toy combinations, creation duration, and hand movement trajectories, establish normative behaviors of sandplay for children of different age groups, and improve assessment efficiency and objectivity. Furthermore, the sand table assessment can complement traditional psychological assessment tools (such as the Wechsler Intelligence Scale for Children), providing a more comprehensive reference basis for children's mental health screening and clinical diagnosis. It is expected to become an important assessment method in school mental health surveys and early intervention for special children.

## 6 Conclusion

Psychological sandplay therapy, with its core advantages of non-verbal nature, fun and creativity, has played a significant role in various rehabilitation scenarios such as children's emotional disorders, autism spectrum disorders, and ADHD. It not only provides a safe psychological expression and emotional release space for children with limited language expression abilities, but also offers multi-dimensional objective evidence for clinical assessment and intervention. Although there are still challenges in the construction of a specialized training system, the formulation of standardized operation procedures, and the unification of evaluation standards at present, with the deep integration of digital technology, the collaborative integration of interdisciplinary therapies, and the establishment of a specialized talent pool, the application value and development potential of psychological sandplay therapy are continuously emerging. In the future, by further improving the standardized assessment system, promoting the implementation of AI-driven intelligent analysis systems, and expanding their application scenarios in school mental health surveys and early intervention for special children, psychological sandplay therapy is expected to become a key supporting measure in the child mental health service system, providing more precise and efficient support for promoting the healthy development of children's physical and mental health.

## 7 Limitations

This is a narrative review, and it has certain limitations in terms of methodology. Firstly, the analysis of the application of psychological sandplay therapy in various child rehabilitation scenarios in the article is mostly based on the integration of existing research results, lacking the support of large-scale, multi-center, prospective clinical control trial data, and the evidence-based level of the conclusions needs to be improved; Secondly, the discussion of the AI intelligent analysis system focuses on the theoretical framework and application prospects, without covering the details such as algorithm accuracy, data annotation norms, and clinical verification results in the specific technical implementation; Thirdly, the design of personalized sandplay intervention plans for children with different disorder types lacks in-depth case analysis, and fails to fully demonstrate the differentiated adaptability of the treatment strategies; Fourthly, the influence of cultural background on the interpretation of sandplay symbolic meaning has not been systematically considered, and the local adjustment paths in cross-cultural applications still need to be further explored. These limitations provide directions for subsequent research and suggest that in the future, empirical research, technical verification, and cultural adaptation analysis should be combined to more comprehensively promote the scientific development of psychological sandplay therapy.
